# “Somewhere along the line, your mask isn’t going to be fitting right”: institutional racism in Black narratives of traumatic brain injury rehabilitation across the practice continuum

**DOI:** 10.1186/s12913-024-10986-1

**Published:** 2024-07-24

**Authors:** Samira Omar, Charmaine C. Williams, Laura B. Bugg, Angela Colantonio

**Affiliations:** 1 Rehabilitation Sciences Institute, University of Toronto, Toronto, Ontario Canada; 2 Factor-Inwentash Faculty of Social Work, University of Toronto, Toronto, Ontario Canada; 3 Global and Community Health, University of California Santa Cruz, Santa Cruz, CA USA; 4 KITE-Toronto Rehabilitation Institute, University Health Network, Toronto, Ontario Canada; 5 Department of Occupational Science & Occupational Therapy, University of Toronto, Toronto, Ontario Canada

**Keywords:** Traumatic brain injury, Rehabilitation, Institutional racism, Narrative inquiry, Critical race theory

## Abstract

**Background:**

Over two decades of research about traumatic brain injury (TBI) rehabilitation emphasized the persistence of racial health disparities in functional outcomes that disproportionately impact Black populations without naming or addressing racism as the root problem. Further, the experiences of Black people with TBI have yet to be documented and accounted for in scientific scholarship from the perspectives of Black persons in Canada.

**Purpose:**

This study intended to examine the rehabilitation narratives of Black TBI survivors, family caregivers, and rehabilitation providers and use critical race theory as a conceptual framework to understand how anti-Black racism manifests in those experiences.

**Methods:**

Through critical narrative inquiry informed by a critical constructivist paradigm and a critical race theory lens, in-depth narrative interviewing were conducted with seven survivors, three family caregivers, and four rehabilitation providers. Data were analyzed using reflexive thematic analysis within and across groups of participants to conceptualize themes and sub-themes.

**Findings:**

Themes captured how racism becomes institutionalized in TBI rehabilitation: (1) the institutional construction of deficient Black bodies, (2) the institutional construction of rehabilitation access, (3) the institutional investment in resisting and approximating whiteness in rehabilitation practice, and (4) the institutional construction of deficient Black futures.

**Conclusion:**

Study findings point to the dire need to ensure rehabilitation programs, services, and the delivery of care are not determined based on inequitable practices, racial biases and assumptions about Black people, which determine who deserves to get into rehabilitation and have opportunities to be supported in working towards living a full and meaningful life.

**Supplementary Information:**

The online version contains supplementary material available at 10.1186/s12913-024-10986-1.

## Background

Traumatic brain injury (TBI) can be described as a chronic disease process that often leads to permanent and persistent challenges to physical, cognitive, and psychological functioning, which require a long strenuous course of rehabilitation [1]. Although TBI can impact any individual, some groups of people, such as Black populations, have a higher likelihood of sustaining injury [2–4] and living with the long-term impacts [5]. Despite the importance of rehabilitation, Black people living with the consequences of TBI not only lack access to rehabilitation services [6–11]; they also experience poor functional outcomes [12] and challenges with community integration [13–16] compared to White populations and other racial minorities. The U.S. Centers for Disease Control and Prevention (CDC) recognized racism as a critical contributor to racial health disparities and expressed a commitment to address the social and structural conditions that bring about these forms of inequities [17]. In the institution of TBI rehabilitation practice, this raises a critical question, “how is racism operating here?” [18].

Considering the mechanisms in which racial disparities persist draws attention to the impacts of institutional racism [18]. Better [19] defines institutional racism as the “patterns, procedures, practices, and policies” that function within institutions to intentionally “penalize, disadvantage, and exploit” racialized persons (p.11). For example, several rehabilitation clinical practice guidelines have been created to inform practice for persons with TBI using the most up-to-date evidence-based research, which provides recommendations for rehabilitation professionals and healthcare providers to promote optimal functioning and recovery post-injury [20, 21]. One form of scientific evidence that can be used to inform these practice guidelines is the first-hand rehabilitation experiences of persons with TBI, which predominately give insight into the organization of rehabilitation services [22], effectiveness of programs [23, 24] perceptions and experiences about the rehabilitation process [25–27], transition into the community [28, 29], gendered [30, 31] and cultural experiences of rehabilitation [32], and service needs [33]. One of the ways in which institutional racism operates is through the silencing and erasure of the experiences of Black people with TBI. Existing qualitative studies provide limited insight into the experiences of Black and other racialized persons with TBI. While literature reports that racial health disparities disproportionately impact Black people with TBI, such qualitative studies either exclude race data in the demographics or aggregate the data to present a collective narrative that tells the story of the TBI rehabilitation journey as though it is a universal one.

In 2020, the Public Health Agency of Canada (PHAC) released the Social Determinants and Inequities in Health for Black Canadians snapshot documenting anti-Black racism as a determinant of health outcomes and a pertinent factor in health and institutional disparities [34]. In a recent scoping review, Omar et al. [35] examined the clinical journey of Black TBI patients to understand better what is known about the care continuum for this population. Amongst many important findings from this review, the most striking was how racism was identified as a normalized practice in research and one that largely remained unnamed but, in some instances, was referred to in other terms such as racial bias, prejudice, and systemic barriers which are considered as components of racism [35] However, the need to examine the first-hand experience of Black persons with TBI, how racism manifests in rehabilitation institutions, and the outcomes for Black persons with TBI remain.

To date, the TBI rehabilitation literature that considers Black populations has primarily focused on documenting racial health disparities in recovery and functional outcomes using methodological approaches driven by positivist paradigms based on decontextualized knowledge that leaves out the contextual reality of Black lives. The findings from these studies suggest that some rehabilitation institutions fail to fulfill their stated purpose of supporting optimal functioning at home and in communities. Critical qualitative health research has the potential to illuminate the fundamental mechanisms that contribute to differential outcomes in the everyday lives of people who experience social injustice daily [36]. As such, an analysis of the rehabilitation experiences across different institutional spaces across the continuum of care is vital in identifying how institutional racism shows up in practice. Using a critical approach can suggest where researchers, rehabilitation professionals, educators, and policymakers can intervene to dismantle the institutionalized practices that contribute to poor outcomes and limited life opportunities for Black people with TBI. Therefore, this study addresses the following research question using critical race theory (CRT) to foreground institutional racism: What do the stories of Black survivors of TBI, their family caregivers, and rehabilitation providers tell us about how racism manifests in rehabilitation?

## Methods

### Study design

This study used a constructivist-informed narrative inquiry [37, 38]. Narrative inquiry provides a window into understanding the experiences of participants and the meanings they attach to those experiences as, "story makes the implicit explicit, the hidden seen, the unformed formed, and the confusing clear" ([39], p.196). This design aligned with the lead investigators' constructivist worldview such that humans carry themselves through the world through storytelling, which provides richness and depth into the multiple lived realities of the participants and how they make sense of those realities [38, 40]. Using a constructivist paradigm allowed the researchers to understand and explore the multiple realities experienced by the participants. Constructivists adhere to a relativist position, meaning they do not believe in one truth but in multiple and equally acceptable subjective realities that differ from one individual to another [41]. Operating from this paradigm also meant that the relationship between the researcher and the participants were transactional, meaning that the findings from the study emerged from knowledge co-constructed by the investigator and the participants [41, 42]. The co-constructive nature of narrative inquiry allows for unique insights into participants' experiences and enables researchers and clinicians to understand how care can be improved [38, 43]. Riessman [38] acknowledges that the stories people live and tell happen in a particular context and are situated historically, socially, and politically, thus, they must be understood within prevalent discourses and power relations. As such, CRT is used as a conceptual framework to understand the rehabilitation narratives of the participants in this study. This methodological bricolage is known to examine the experiences of people who have been marginalized, silenced, and disempowered [44]. This study was approved by the Research Ethics Board at the University of Toronto (protocol # 40525). This manuscript was prepared using the consolidated criteria for reporting qualitative research (COREQ) checklist [45].

### Theoretical framework

This study was situated in a CRT lens which is a framework that provides opportunities to examine pertinent issues of race and racism by considering the role of institutions by drawing on the experiences of groups of people who are deeply impacted [44]. CRT proceeds with an understanding that although institutional racism is less visible than individual forms of racist actions, the effects can be more harmful as they require change beyond an individual and establish the norms that govern institutions and organizations. CRT emerged in the mid 1970’s to address the impacts of race and racism in the U.S. judicial system [46, 47]. There are five basic CRT tenets that are widely used by theorists. First, racism is a pervasive feature of society that operates through colourblind ideologies that evade race and its contributions to treatment and outcomes [48–50]. Colourblindness is based on the belief that the racial group to which one belongs and any differences based on race should not be considered in decisions [48–50]. Second, race is a social construction, a product of social and political ideas that influence how people relate to one another [49]. This means that race is not biological; instead, it is a social classification system created by humans [49]. Third, whiteness is a form of property that grants White people a set of privileges based on their identity [51]. Fourth, the concept of interest convergence explains that any progress that is made to address racial health disparities, will happen as long as it benefits the dominant majority, which is White people [49]. Fifth, taking action on systemic racism requires counternarratives to the deficit narratives typically represented in research and practice [44, 49]. Based on these five tenants, CRT serves as a suitable framework to examine the impact of race and racism in the institution of rehabilitation facilities for Black people with TBI. These manifestations of these CRT tenants and concepts are described in more detail in reporting the qualitative findings and are further elaborated in the discussion section.

### Participant recruitment

Participants were recruited using purposeful sampling [52] to find Black survivors of TBI, family caregivers, and rehabilitation providers who had knowledge and experience with TBI rehabilitation in a Canadian context. This involved directly approaching organizations and spaces which have people who have knowledge about the scope of the study. Snowball sampling was also employed by asking those recruited who reached out to share the study information with other potential participants such as family, friends, and co-workers who meet the criteria to participate [53]. Survivors of TBI and their family caregivers learned of the study through rehabilitation providers in their circle of care who facilitated recruitment by informing potential participants of the study. Recruitment flyers and messages were shared on Twitter including announcements made in newsletters from local and national brain injury and rehabilitation organizations. Rehabilitation providers were informed about the study through local brain injury and rehabilitation organizations across Canada, professional practice associations who shared recruitment flyers through weekly newsletters and emails to members, and on Twitter.

All persons were required to self-identify as Black or someone from the African diaspora, communicate in English, and be consenting adults capable of providing informed consent to participate in the study. Table 1 provides a summary of the inclusion criteria for all categories of participants. Information about TBI was gathered using the OHIO State University Traumatic Brain Injury Identification Method (OSU TBI-ID) which captures details about their injuries and history [54]. Interested participants contacted the first author, who provided detailed information about the study and confirmed their eligibility. All participants were emailed a research package including consent forms, interview questions, and instructions on connecting virtually using the Zoom platform. Informed consent was obtained from all participants before the start of their interview. Providing questions ahead of time was important to help address the power differentials in the research and to empower participants by informing them of what they will be asked as well as giving them the necessary support.

**Table 1:** Inclusion criteria for study participation

Inclusion Criteria	
**Category of Participant**	**Inclusion criteria**
Person with TBI*	Received or currently receiving rehabilitation services (e.g., occupational therapy, physiotherapy, speech-language pathology, or social work) across Canada Self-identify as someone with a TBI or diagnosed with one which was confirmed using the OHIO State University Traumatic Brain Injury Identification Method (OSU TBI-ID)
**Family Caregiver**	An unpaid family member or friend providing support with activities of daily living for someone who is Black and is experiencing or living with a TBI in Canada OR, community advocate and/or spiritual leader, and others working for and with Black communities and holding expertise and lived experience of TBI
Rehabilitation Provider*	Paid rehabilitation care provider such as a rehabilitation nurses, occupational therapists, physical therapists, speech-language pathologists, social workers, and/or a personal support worker working across the care continuum (ex: in-patient, outpatient, community)

^*^All participants received some or all four of the following services, occupational therapy, physical therapy, speech-language pathology, and/or social work

^*^Rehabilitation providers had to have experience working with visibly Black TBI patients

### Data collection

Demographic information was collected from all participants at the beginning of the first interviews including self-identified ethnicity, racial identity, age, place of birth, gender identity, religious practice, marital status, level of education, place of residence, place of employment, and household income. Specially for persons with TBI, demographic questions were asked about their injury, satisfaction with rehabilitation, the kinds of services they received, and how they paid for these services. Family caregivers and rehabilitation providers were asked similar questions from their perspective. As outlined by Bertaux and Kholi [55], two-step narrative interviewing (e.g., extensive narrative and a period of questioning) was used as the primary method to co-construct narratives with the participants as a form of data. This required creating an environment that enabled active listening and probing about participant narratives. The first author led these narrative interviews and conducted them virtually in a private office at the University of Toronto. Interviews with persons with TBI were spread across three days as part of the empathetic interviewer-participant relationship [56]. One survivor attended the interview with a family caregiver and rehabilitation provider present. At the beginning of the interview, all participants were reminded of the goal of the study to provide context to the interview questions they were asked. The primary question was, "can you please tell me in as much detail as you can your story of what it is like to be a Black man or woman who is living with a traumatic brain injury and your experiences with rehabilitation?" This was followed by a series of open-ended questions that explored participants' narratives based on their responses to the first question and others related to their identities and experiences in rehabilitation. An example of the interview guide used for survivors of TBI can be found in supplementary materials (Additional file 1). Family caregivers and rehabilitation providers where asked similar questions related to their perspective. The second interview, which occurred between one and three weeks apart from the first, was used to probe the experiences that participants shared in their first interviews. At the end of the second interview, all participants were asked, “what is the final message you wanted to get to me about the Black experience in this space?” In total, fourteen participants (ten women and four men) were interviewed, and each interview lasted, on average, between 60 to 90 minutes and were audio-recorded. The final sample size was nuanced and rich in data to capturing similarities, nuances, contradictions, and tensions among the amount of detail that participants provided about their experiences of being Black in TBI rehabilitation [57, 58].

### Data analysis

Data were analyzed in this study using reflexive thematic analysis [59–61] and informed by CRT. Reflexive thematic analysis serves to identify patterns and themes within and across participant’s narratives of their rehabilitation experiences. Reflexive thematic analysis was chosen due to its (a) alignment with the methodological approach of narrative inquiry and the goal of identifying common patterns, ideas, and themes across the transcripts while staying close to participants’ own words; (b) theoretical flexibility to bring in paradigmatic and epistemological positions aligned with the study design; and (c) suitability for applied health research with social justice implications for practice [61, 62]. Reflexive thematic analysis was used inductively in this study which means that analysis of narrative interviews was grounded in the data [61].

The steps taken to conduct this analysis were guided by Braun and Clarke’s [59, 61] guidelines for using thematic analysis. This analysis was focused on showing how racism becomes an institutionalized practice in TBI rehabilitation. The audio-recordings of the interviews were transcribed verbatim by a professional transcriptionist. Data familiarization was undertaken by the first author who re-listened to all the audio-recordings of the interviews and did multiple and iterative readings of the transcripts. This step involved writing reflexive notes about common patterns and differences, contradictions, and tensions amongst and across the different interview transcripts. As transcripts were read, initial codes were written alongside the margins of transcripts. Open coding was used to generate initial codes using analytical strategies such as asking questions of the data in line with CRT and the focus the study. Visual diagrams of the codes and patterns of the data were created at the beginning to get a more global sense of the generated codes. Weekly meetings were held with the research team to discuss identified codes, patterns, and reflections that were developed during the analysis process. This was an iterative process which involved hand drawn visual schemas where the first author moved back and forth between different phases of analysis outlined in Braun and Clarke [59, 61] until the story being told about the data became clearer. Once preliminary themes were identified and codes were refined, the first author then transferred all data onto NVivo 12 where the transcripts of rehabilitation providers and family caregivers were also coded separately using the identified pre-liminary themes and codes from the survivor transcripts. Given the unexplored nature of these voices, overarching themes were developed with the entire dataset in mind [59, 61]. A final thematic diagram was produced and continuously modified during the writing stage to demonstrate the relationship between the themes and the findings. All steps of the coding and theme development were thoroughly documented to ensure rigor and trustworthiness.

### Reflexivity: researcher as an instrument

Finlay [62] defines reflexivity as a "thoughtful, conscious self-awareness in research" (p.532) through an ongoing and dynamic evaluation of the research process and the subjective nature of doing qualitative research. Researcher subjectivity was considered an integral resource in this study, and reflexivity was integrated as part of the dynamic of the research team and the co-construction of knowledge throughout the process [61]. For example, during the interviews, this guided the lead authors’ judgement in how to respond to participants and the types of experiences to probe. The researchers in this study came in with a conscious awareness of how Black people have been exploited and harmed historically in research studies. An integral part of co-constructing knowledge with the participants involved the lead researcher sharing her insider knowledge. As a researcher, it was important for her to be immersed in the research and share with participants how she related to this work to build trust, which led to the kinds of stories shared in the interviews [63, 64]. This trust was built before starting the interviews and as part of the informed consent process where the lead researcher was explicit about the intentions behind doing this study, how she related to the participants, how their data was going to be stored and used, and the confidentiality of their identities. Building rapport with the participants led to the production of richer data which allowed participants to enter into a space where they could share these profound experiences more easily [65].

The research team is composed of individuals with diverse disciplinary backgrounds and methodological experiences, which shaped the study design, execution of the interviews, and data analysis. Members of the research team engaged in collective and one-on-one meetings with the lead researcher throughout the research process. The lead researcher documented the tensions between navigating insider and outsider perspectives, the power dynamics at play, and how this influenced the co-construction of knowledge with the research team in her reflexive journal. In this journal, she also recorded insights, comments, conversations, and emotions that emerged and evolved throughout the research process. Before, during, and after interviews, she wrote personal reflexive observations about how her insider and outsider perspectives shaped and influenced the research process (e.g., research-participant relationship and interactions). Methodological reflexivity was also practiced, where the researchers played particular attention to why decisions were made around the nuances of choosing an approach to analysis (e.g., reflexive thematic analysis vs narrative analysis), using a conceptual framework, selecting particular quotes, explaining the context surrounding the quotes, and how these aligned with the nature of co-constructing knowledge to ensure research rigor and ethical considerations [66, 67].

### Quality criteria for methodological rigor and trustworthiness

Methodological rigor and trustworthiness of the findings were maintained through various criteria outlined in Tracy [67] and described below. Triangulation of data [68] was used in two different ways in this study, including through the various approaches to collecting data such as demographic questionnaires and narrative interviews and through the triangulation of different study participant perspectives, such as persons with TBI across the spectrum of injury, family caregivers, and various rehabilitation providers. Investigator triangulation was used to minimize the potential bias of only having one person code, gather, and analyze the data [68]. The divergent viewpoints of members of the research team were used to cross-check and verify the interpretation of data by other members of the research team through regular discussions and meetings. Periodic peer debriefing [69] with critical friends such as the doctoral supervisors, committee members, and knowledgeable qualitative health researchers was used to mitigate the different ways the researcher influenced the research process. As part of peer debriefing, the first author engaged in regular meetings with critical friends before and after interviews to share, discuss and challenge ideas throughout the analysis and writing process. Prolonged engagement and immersion with the data through data collection and analysis [70, 71] was applied, and through this, the first author spent long periods with participants to build rapport which led participants to open up more during the interviews. Reflexive notes were recorded throughout the process. The audit trail was maintained through different stages of the research process, including the conception of the study, generation of codes, construction of themes, questions raised about the connection between themes and codes, and tensions and contradictions raised amongst different participant experiences. Lastly, the credibility of the results was established through thick descriptions using detailed quotes.

## Results

A total of seven survivors of TBI, three family caregivers, and four rehabilitation providers across the province of Ontario participated in this study. Table 2 provides the socio-demographic characteristics for all participants. Of the sample of survivors, most identified as sustaining moderate to severe TBI (57%) and were of ages ranging from 19 to 52 with an average age of 35. 3 ± 9.4 years. Time since injury of the most recent TBI ranged from as little as one year to 17 years since the most recent TBI. Information about sex (e.g., male and female) was collected. However, participant gender is only reported in the demographic table. Women made up 57% of the sample of persons with TBI. A total of 29% of these participants sustained their injury through motor-vehicle collisions, another 29% through bike accidents, and one through an assault. Two individuals sustained their injuries during the COVID-19 pandemic, and all persons with TBI were currently receiving some form of rehabilitation for their injury (See Table 3 for injury characteristics). There were three family caregivers who identified as women, with an average age of 45.7 ± 4.5 years and spent an average of 3.3 years caregiving for their loved ones. Two were caregivers to their children with TBI (son and daughter) and one was a caregiver for her husband. Lastly, two physiotherapists, one occupational therapist, and one social worker participated. About 75% of the rehabilitation therapists were women, with an average age of 47 ± 13.1 years. Years of experience ranged from as little as five years of experience to over 30 years. Table 4 provides more information about the practice settings and years of experience for caregivers and rehabilitation providers.

**Table 2:** Sociodemographic characteristics for survivors of TBI, family caregivers, and rehabilitation providers

**Participant Categories**	**Survivors (n = 7)**	**Family Caregivers (n = 3)**	**Rehabilitation Providers (n = 4)**
**Socio-demographic Characteristics**	n (%)	n (%)	n (%)
**Self-identified Nationalities**			
African	6 (85.7)	2 (66.7)	4 (100)
Caribbean	1 (14.3)	1 (33.3)	
**Age**			
18-29	1 (14.3)		1 (25)
30-49	5 (71.4)	1 (33.3)	1 (25)
50-64	1 (14)	2 (66.7)	2 (50)
**Gender**			
Men	3 (42.9)		1 (25)
Women	4 (57.1)	3 (100)	3 (75)
**Highest Level of Education**			
Some post-secondary	2 (28.5)	1 (33.3)	
Bachelor’s degree	2 (28.5)		
College diploma	3 (43)	2 (66.7)	1 (25)
Master’s degree			3 (75)
**Occupational Status**			
Unemployed or on leave	5 (71.4)	1 (33.3)	
Employed	2 (28.5)	2 (66.7)	4 (100)
**Marital Status**			
Single	5 (71.4)	2 (66.7)	1 (25)
Common Law	1 (14.3)		
Married	1 (14.3)	1 (33.3)	3 (75)
**Number of Children**			
None	6 (86)		2 (50)
1-3		1 (33.3)	2 (50)
4-6	1 (14.3)	2 (66.7)	
**Household Annual Income**			
Not disclosed	1 (14)		
<$30,000	2 (28.5)		
$31,000-$69,999	3 (43)	1 (33.3)	
$70, 000 - $90, 000		1 (33.3)	
>$100,000	1 (14.3)	1 (33.3)	4 (100)
**Self-identified Religious Affiliation**			
Spiritual	4 (57.1)	1 (33.3)	
Christian	2 (28.5)	2 (66.7)	
Catholic	1 (14.3)		
**Current Living Arrangement**			
Living alone	5 (71.4)		1 (25)
Living with family	2 (28.5)	3 (100)	3 (75)

**Table 3:** Injury characteristics for survivors of traumatic brain injury

**Characteristics for Survivors of TBI (*n* = 7), n (%)**	
Self-Identified Injury severity*	
Mild	2 (28.6)
Moderate	1 (14.3)
Moderate to Severe	4 (57.1)
Mechanism of Injury*	
Motor-Vehicle Collision	2 (28.6)
Pedestrian	1 (14.3)
Bicyclist	2 (28.6)
Assault	1 (14.3)
Workplace Injury	1 (14.3)
**Time Since Recent Injury (Years)**	
1-9	5 (71.4)
10-20	2 (28.6)
**Injury Sustained During Ongoing COVID-19 Pandemic**	
Yes	2 (28.6)
No	5 (71.4)
**Lifetime History of TBI**	
1-2	5 (71.4)
3-5	2 (28.6)

Participants self-identified as someone with a TBI or diagnosed with one which was confirmed using the OHIO State University Traumatic Brain Injury Identification Method (OSU TBI-ID). Persons with TBI also self-identified the severity level of their injury as mild, moderate, or moderate to severe. Mechanism of injury represents most recent TBI. The OSU TBI-ID was also used to confirm lifetime history of TBI as well as other information captured about participants history with TBI.

**Table 4:** Caregivers and providers represented

**Caregivers and Providers Represented**	**Caregiver (*n* =3)**	**Rehabilitation Provider (*n* = 4)**
	n (%)	n (%)
**Occupational Practice**		
Program officer	1 (33.3)	
Administration	1 (33.3)	
Business owner	1 (33.3)	
Occupational therapy		1 (25)
Physiotherapy		2 (50)
Social work		1 (25)
**Practice Setting**		
Private Practice (e.g., community, in-clinic)		3 (75)
Education Setting		1 (25)
Government	2 (66.7)	
Corporate	1 (33.3)	
**Years of Experience**^a^		
1-5	3 (100)	1 (25)
>20<30 years		1 (25)
>30 years		2 (50)

^a^Years of experience for caregivers based on years of caregiving for person with TBI

**Fig. 1:**
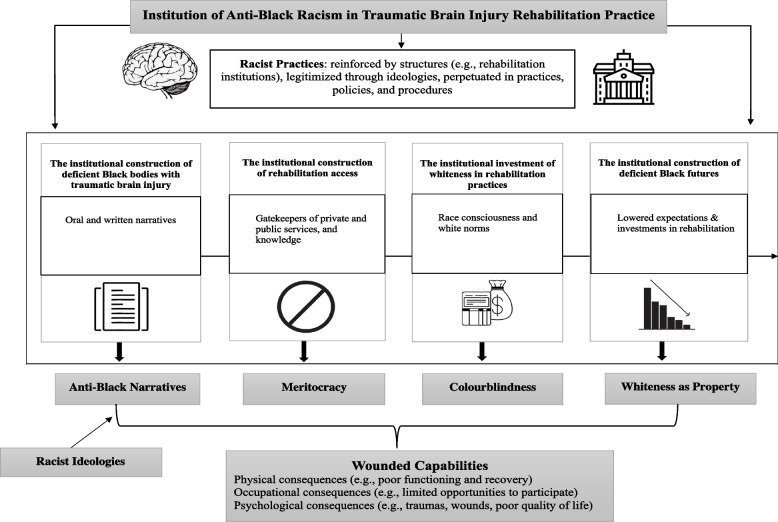
Institution of anti-black racism in traumatic brain injury rehabilitation practice

It is important to note that the knowledge produced in these findings were co-constructed between the researcher and participants during the narrative interviews. In the reporting of the findings, the reader will be hearing from the participants but also from the lead investigator as she brings forward the process, she was in with co-constructing these narratives. Four themes and eight sub-themes were conceptualized, describing how racism becomes institutionalized in Black experiences of TBI rehabilitation. Figure 1 depicts a pictorial representation of the relationship between the conceptualized themes and CRT ideas. The themes revealed that racism becomes institutionalized in the practices, procedures, and policies perpetuated and upheld through rehabilitation providers in four different ways: (1) the institutional construction of deficient Black bodies, (2) the institutional construction of rehabilitation access, (3) the institutional investments in resisting and approximating whiteness in rehabilitation practice, and (4) the institutional construction of deficient Black futures. Below, these findings are presented.

### The institutional construction of deficient Black bodies “unworthy” of rehabilitation: “Black people are seen, just like a piece of meat, just baggage” (P14)

This theme focuses on how rehabilitation providers and other members of the care team in the institution of rehabilitation construct deficient Black bodies through oral narratives shared between rehabilitation provides and narratives documented into rehabilitation practice. This theme is further divided into two sub-themes: a) oral narratives shared between rehabilitation providers and b) written narratives documented into medical records.

#### Oral narratives shared between rehabilitation providers

The narrative surrounding a particular patient can determine who is worthy of rehabilitation, in the process producing differential treatment and outcomes. Participants’ narratives reveal that there are two types of TBI patients: (a) the ideal patient who is white (regardless of gender), experiences manifestation of their injury which other service providers and members of the rehabilitation team attribute to the injury itself and are deserving of rehabilitation and (b) the problematic Black patient who is aggressive, violent, dangerous, and exhibits behavioural manifestations of the injury but is perceived as being “unmanageable” and undeserving of rehabilitation. These are the oral narratives that get institutionalized about Black bodies with TBI.

These oral narratives that are circulated about Black TBI patients in clinical settings shaped rehabilitation encounters and the quality of care received by Black survivors. Participants shared that Black TBI patients who were men were seen as more violent, dangerous, and aggressive as part of their baseline functioning. One caregiver described it as the following, *“They're bringing in their own prejudiced judgement and subjectiveness in it, instead of remaining objective, being professional, being courteous, treating people with dignity, irrespective of their socioeconomic status, background. You know, treating them like they're a human being”* (P16, Woman, Family Caregiver).

One rehabilitation provider shared a contrast between how Black caregivers were seen by the clinical team when they advocated for their loved ones compared to White caregivers. She described, *“Why is it that when black people advocate for their loved ones that it’s seen that these family members are being difficult, are challenging, are hard to work with? White families are seen collaborative, working in partnership, being invested in their loved one’s care you know? Black caregivers are being given all those negative titles being problematic, being troublemakers, being difficult…”* (P14, Woman, Rehabilitation Provider).

One caregiver shared the following when she talked about how she was perceived by members of her daughter’s rehabilitation team as a Black woman who was from a visible religious background, *“If we get assertive – because with me too. I remember one time I was told there’s no need to get upset. I wasn’t getting upset, I was elevating my voice a little so that you can hear me, because it seems like the person couldn’t hear what I was saying…I knew right then and there, that comment alone told me, you know, you're looking at me, I'm a black woman, and you think that all black women are angry, bitter women. It’s that stereotype…”* (P5, Woman Caregiver).

Rehabilitation providers shared that when Black male patients displayed behavioural outputs related to their TBI, *"…there is less maybe tolerance for it, less understanding for it, less realizing that this is related to the brain injury, this is not necessarily who they were before. I think that there’s a lot of assumptions that this is just the person and when that person is not black, I have seen a lot more tolerance to it. Of, oh that’s just a brain injury”* (P1, Woman, Rehabilitation Provider). Another rehabilitation provider described, “*…the person could be acting the exact same way [white patient], and then they're not - they won't say that they're aggressive. And so, you know, they just, they don't get that it will affect the type of treatment that they get, as well… basically people are, you know, stereotyping or just jumping to these conclusions as well, without actually, you know, getting to know the person*” (P12, Man, Rehabilitation Provider).

In rehabilitation for Black male TBI patients, participants perceived that the etiology of injury served as a mechanism to reinforce racial profiling in rehabilitation by way of assumptions of criminality and being unworthy of care. Some of the female rehabilitation providers shared, *“…for blacks in general, there’s a lot of stereotyping, and biasing, and so people already come in with assumptions. And then once you’re coming in, and you were, well, this is the result of assault, I think there’s a lot of assumptions that go with that about who you are, how you are, how you’re treated…”* (P11, Woman Rehabilitation Provider). All they saw was that this person *“…must have been a bad person to get that injury. And they don’t even sometimes try to make the connection as a human being to that person… And that along with the implicit bias that all black people are violent or aggressive…”* (P1, Woman Rehabilitation Provider).

Again, that is not to say that the etiology of injury creates these harmful narratives, rather they reinforce existing narratives that Black TBI patients, particularly males are dangerous, aggressive, violent, and a threat. These racially biased narratives have important implications as they influence how other service providers treat the Black TBI patient based on participants’ experiences which have been shown to be harmful during clinical encounters with other rehabilitation team members and is illustrated in the extract below.

*“… trust me every time that [behavioural issue] happens and I’m involved with that person, they’ve already talked about the person being violent, if it’s a black person. Even if you step back and you go well, they have a brain injury…But, you know, I don't know if you've heard the cliche that as long as you are black and you are male you are a threat to the world, right? And essentially…don't ever have a brain injury in the hospital and be aggressive because they're going to come down on you, hard, right? And this guy was being treated as if he – I mean he had a brain injury and he did have behavioural issues…And because I deal with brain injuries specifically there are ways to deal – and he's in a rehab setting that supposedly deals with brain injury but they still didn't get it. You don't have a million people going through somebody's room with all different ways of working with him and expect him to be OK. He's just come out of his accident, he's just woken up, he's got some major, major behavioural issues, there should be a way to deal with that person, a specific way that we know how it works but it's not followed through. So then he gets angry and he can sense, even though he's got his brain injury, he can sense the people that don't really want to be there, right? So he gets his – his behaviour becomes bad because he knows that person is going to treat him not very well…but he's black so he's just violent, right?* (P1, Woman, Rehabilitation Provider*).*

This rehabilitation therapist expressed her concerns that all Black male TBI patients are always referred to as violent the moment they present behavioural problems related to their brain injury. These are the kinds of stereotypes that are portrayed about Black men as inherently being criminals and dangerous which reinforces their criminalization [72]. As illustrated in the extract above, when the rehabilitation provider states, *“…he's in a rehab setting that supposedly deals with brain injury but they still didn't get it. You don't have a million people going through somebody's room with all different ways of working with him and expect him to be OK…”,* other members of the rehabilitation team did not follow protocol and actually work to help the individual defuse the behavioural outputs in a manner that was appropriate for someone with a TBI, particularly someone who was assaulted. By rushing into his room in large numbers, this could re-traumatize and trigger the patient. The rehabilitation provider also indicated something important by revealing that she noticed his behaviour would get worse when he knew was under the care of other providers who would not treat him like a person suggesting that the behavioural outputs might be his way of communicating that he was in danger. From the reporting of these encounters, rather than taking the time to consider what triggers a Black TBI patient, rehabilitation therapists and other service providers would only consider the fact that a Black person with TBI is just a Black person and that all Black men are violent and aggressive. This suggests that Black TBI patients who are seen as being unmanageable and violent at their baseline are not seen as people who can benefit from rehabilitation and, therefore, will not be recommended for opportunities for recovery and regaining functioning. For the patient displayed in the example above, the clinical setting was so bad that he had to transition out of the hospital sooner as the space was no longer considered to be safe for him.

Additionally, in a rehabilitation setting, these deficient narratives also resulted in Black TBI patients being discharged sooner and disconnected from rehabilitation. For this patient represented in the example below, he shared that there was already an oral narrative surrounding him being a threat, dangerous, and violent based on his appearance as a tall Black man with a TBI. In rehabilitation, he described many instances where they treated him like he did not have a TBI and racially profiled him like a criminal. He explained that at one point, he was nearly assaulted by security guards as an in-patient and had to call his mother to protect him from what harm could have happened, which he shared was a traumatizing experience for him and his family. This was the narrative that other service providers told about him; after that incident, he was discontinued from rehabilitation. In two separate interviews, a male survivor and his occupational therapist recounted this experience and shared,* “…I had an episode, because I don’t know what triggered it, but I know it had to do with the pharmaceutical drugs, what they’d been giving me*” (P15, Man Survivor). He described that there was no understanding *“they didn’t have anything to see to it, you know, to see that, OK that was a guy who had a traumatic brain injury, you know, as a result of of of the event*” (P15, Man Survivor).

The occupational therapist described that his rehabilitation *“…was cut short because of what happened, and then COVID also happened around the same time as well”* (P12, Man Rehabilitation Provider).

#### Written narratives documented in medical records

Another way that the institution of rehabilitation practice constructs deficient Black bodies is through oral narratives that become documented in medical records. One rehabilitation provider shared a narrative about what he would often notice would happen to Black male TBI patients he would be working with and the kind of narratives that he would see get documented into their medical records which he shared had implications for how other providers would see him. He described,

*“I’m reading his medical records and you see these diagnoses; I start seeing these psychiatric diagnoses. And then to hear, like, from the client it’s like, well, they –that’s just the diagnosis that they gave me. It’s not true or and then to hear that it’s very tough, especially as a therapist. Because those are – that is kind of the reality, those stories that we hear all the time…That’s also something I would never document as a weakness as a therapist just kind of once I speak with the client more and get a bit of a more information from them on that side of it. And I would never document that as a weakness where I’ve seen it documented as a weakness. So I think that’s really also too that’s a huge part as well is dealing with other therapists and then what they’re putting in their notes in their records”* (P12, Male, Rehabilitation Provider).

White TBI patients were not only tolerated and given patience in the clinical setting but there was also careful consideration of what narratives about them were shared and documented in their medical charts. For example, one rehabilitation provider shared her encounter with a White male TBI client. The following was her experience,

*“…Nobody ever told me that this guy was touch intolerant, like if you put your hands on him, he doesn't like it and that's all I was doing. Nobody ever told me that he had beaten up every single girlfriend he was with, violence. Nobody ever told me that he spent his whole working life fighting people, lost lots of jobs because he was aggressive to his co-workers and then I'm going in there as a black person, [patient] don't like black people. And I didn't go back…But when I told her [boss] about this incident she said to me are you sure you're not just being too touchy. And I go you know what, if you think you can get me to go back to see him, you're going to have to think again…”* (P1, Woman, Rehabilitation Provider).

She described that she was expected to provide physical therapy to this White TBI patient despite his tendency to be violent and uttering death threats to other service providers about what he would do to her as a Black woman. The excerpt above also suggests that other service providers were aware of these narratives, but they were not documented in his medical records. By saying, *"…are you sure you're not just being too touchy…"* her boss made excuses that took the focus away from the White patient being violent and instead placed the blame on the Black woman provider, also implying that the behaviour is attributed to the brain injury and not him as a person. This illustrates that whiteness affords White TBI patients a level of protection from the harmful implications of written narratives of violence and aggression, reinforcing who deserves rehabilitation. Some of the rehabilitation providers did not explain, rather they went right into showing the comparison knowing that as a Black woman whose been in this space, there was a shared understanding of the comparison being made. In the excerpt above, what is unspoken but was communicated as part of the encounter in the interview was that a comparison was being made here between what was happening with this patient and what would happen if the patient were Black which is a comparison that was similarly expressed by other female providers. Beyond the portrayal of Black TBI bodies as deficient, this also provides a window into how the bodies of Black women providers are disregarded and the lack of protection that they receive by not sharing this pertinent information including the employment discrimination [73, 74].

For Black patients, "*the stereotyping is something that really affects what they think of the client and what their recommendations are. And then whatever it is that they put in their notes, that then affects the client during the next steps of the rehab process..."* (P12, Man, Rehabilitation Provider), impacting future possibilities in rehabilitation.

### The institutional construction of rehabilitation access: “Gatekeepers” of rehabilitation treatment (P2)

This theme focuses on the gatekeeping of rehabilitation services describes how rehabilitation professionals are implicated in gatekeeping which limits access to rehabilitation services for Black TBI patients. This theme is divided into three sub-themes depicting the different levels that rehabilitation professionals gatekeep: (a) rehabilitation professionals gatekeep at insurance companies, b) rehabilitation professionals gatekeep publicly funded rehabilitation services, and c) rehabilitation professionals gate-keep knowledge and resources.

#### Rehabilitation therapists gate-keep at insurance companies

One type of gatekeeping described by participants was at the level of insurance companies where rehabilitation providers were hired to determine whether a person qualifies and is deserving of rehabilitation. This was the case for participants who were either pedestrians hit by a moving vehicle or in a motor vehicle collision. All participants who were entitled to insurance coverage had to go through extensive assessments and evaluations to determine their eligibility for rehabilitation funding, and this began with the medical doctor who argued about the injuries sustained by the individual. The narratives of Black survivors and their caregivers revealed how institutional racism manifests in the everyday practice of rehabilitation service providers hired through insurance companies that served to determine whether they get access to finances to fund their rehabilitation. These individuals reported that their experiences felt racially motivated.

For example, one caregiver described an encounter she witnessed when her husband was being assessed by the insurance company to determine whether he qualified for rehabilitation services and if his injuries were related to his TBI or prior functioning. She shared the following interaction with a social worker who was hired by the insurance company to conduct assessments about his level of functioning,

*“[…]“Were you ever diagnosed with a disability or learning disorder? “And he said no, and then she followed up with it, “So school wasn’t hard for you? Like you never found school hard? He’s like, “I was a pretty average student.” And then she’s like, “Oh, so you did good in every grade always in school?” Like she was trying to lead him down a path to give some sort of – to fit into some stereotypical mode so that she has of what the Black experience is growing up. I even spoke to our counsellor about it that sometimes does those kinds of assessments, and she’s like, “They ask about disabilities and childhood trauma just to kind of get an idea of your mental health, where you stand,” and then I explained to her how she kept rephrasing the question and kind of refusing to accept that it was a no. And she’s like, “No, that’s wrong. That’s 100 percent wrong, she should never do that. That sounds racist.” I said, “Well, better you say it than me.” Our counsellor is not Black, but she could definitely recognize it”* (P10, Woman, Caregiver).

In the example above, the participant perceived that the social worker racially profiled this Black man and assumed prior deficiency in functioning attributed to his race through the line of questioning that was asked. This is similar to the body of literature which describes that Black people are labelled as having learning disabilities, lower IQs, are placed in special education classes, and have lowered school performance as a consequence of unscientific discourse [75, 76].

In a separate interview, her husband further described how the social worker was attempting to prove that because he is a Black man, he likely came from a family where the father was violent and abusive towards his mother,

*“[…] her line of questioning geared towards race was inappropriate. It was more along the lines of trying to get a history of my background to depict that because I am a black male that I experienced some form of childhood trauma, which is why my brain injury shouldn’t be justified. And when I say questioning along the lines of, did I ever witness my father abuse my mother, did I ever witness any major physical altercations between family members or friends’ things along that nature which I felt was really targeted towards my race not necessarily my upbringing, which is yeah not cool”* (P7, Man, Survivor).

She was attempting to prove that because he is a Black man, he likely came from a family where the father was violent and abusive towards his mother which draws in the literature about the trope of absent Black fathers and their endangered sons [76, 77]. In the interview encounter, he described that the insurance company denied him treatment three times even though he was officially diagnosed with a TBI by his care team. What is particularly interesting about his experiences and those of all other survivor participants is how they were constantly denied access to rehabilitation funding and services which lead to declines in functional gains, rehabilitation progress, and poor emotional and psychological well-being. Survivors who were covered by insurance expressed that they would get poor quality care where the clinician would cancel an appointment, cut an appointment short, or continue to reschedule but billed for these sessions, which reduced their funding and in many other instances they described how they would be paying for services to get treatments that they did not believe they needed.

#### Rehabilitation professionals gatekeep publicly funded rehabilitation services

Another form of gatekeeping is at the level of publicly funded rehabilitation services, where service providers make decisions and recommendations about who gets to access different rehabilitation programs and services. Participants discussed how being Black was a barrier to accessing different rehabilitation services. Regardless of coverage, survivors of TBI shared that they often felt invisible throughout their rehabilitation journey and described several encounters where they were overlooked, not taken seriously, ignored, neglected, dismissed, and, as a result, were provided treatments that did not meet their needs. For many of these survivors, these compounding experiences of being invisible made them feel depressed because they were not getting the rehabilitation support, they needed to meet their rehabilitation goals and participate in meaningful occupations. For example, one female survivor expressed throughout her entire interviews the countless number of times she was denied entry into different publicly funded rehabilitation programs by rehabilitation professionals and other members of her care team who expressed it was more important for her to work on her mental health than meet other rehabilitation goals necessary for overall well-being. She shared the following,

*“…I’m so disappointed in healthcare system and the people that are put in place that's supposed to help you and then they don't help you… I was sent to, to get outpatient help. And I went there and said, OK I need an ABI case manager, TBI case manager to help me access community resources, to help with housing, this and that…I’ve been waiting almost 10 years, what's going on? “Oh, we [rehabilitation team] decided that that's not what you need, you need to address your mental health. That's why we think that you should go to – you should continue to go to the hospital base, support group to manage depression, anxiety, all the trauma…I said, “Trauma, depression, anxiety is always going to be part of my life. That's not what I need. I need, “Oh, but that's the decision we made, I say, “You cannot make a decision about me without telling me about it”. And all this time, I'm waiting to get somebody to help…I feel very discouraged, because of course, anyone that went through what I went through, will be depressed. But when I finally get to go to [institution name], my goal was rehabilitation. I need to have some kind of routine to help with the mobility activities of daily living. I need that routine. Because if I have a routine and I'm doing all this physical activity to help with walking, doing dishes, mobility, balance, and stuff, of course, I'm going be less depressed.”* (P4, Woman, Survivor).

What is alarming and not surprising is how she self-advocates for herself in expressing the importance of meeting her rehabilitation goals to support her mental health but is ignored by her rehabilitation team. The participant perceived that the rehabilitation team pathologized her by over-emphasizing her mental illness without considering the physical and cognitive aftermaths of the TBI that she had to live with, which impacted her day-to-day functioning and mental well-being. This draws attention to other scholarship which have reported that Black people are more likely to be misdiagnosed for mental illness by White professionals [78] and similar to the ways in which Black patients’ pain levels are less likely to be taken seriously than White patients and other ethnic minorities [79]. This excerpt illustrates how the rehabilitation team made decisions on her behalf without even consulting with her and neglected her input, limiting her capacity to make decisions about her care and suggesting that they knew what was best for her, which was also seen in other interviews with survivor participants. The lack of attention to her physical limitations related to the TBI strained her mental health. In another part of her interview, she described how she attended a hospital-based mental health support group and was traumatized by the experience. This set her back months with her rehabilitation goals because she was exposed to other people’s problems that were overwhelming and detrimental to her mental health. In fact, the gate keeping of publicly funded rehabilitation services by weaponizing the need for mental health was a common occurrence in the narratives shared by some of the other survivors.

#### Rehabilitation professionals gatekeep knowledge and resources

Lastly, rehabilitation providers are also implicated in gatekeeping at the level of knowledge transfer. The interviews of rehabilitation therapists revealed how Black TBI patients, and their caregivers were “short-changed” and not offered or informed of rehabilitation services they were qualified to receive. Rehabilitation therapists assumed Black TBI patients, and their families knew how to navigate an already complicated healthcare system. This is best illustrated in the excerpt below,

*“…but the other thing is it works the other way, so people will see people of colour with locks, hijab, they may not be highly educated, but that doesn’t mean to say that they don’t deserve the same amount of care that somebody else will get. So, there’s those biases that I know happens. And people assume that you know you don’t want to know the information, so they give you the bare bones. And sometimes when you ask a question they go, they like don’t want to give you the answer.”* (P11, Woman, Rehabilitation Provider)

### The institutional investments in resisting and approximating whiteness in rehabilitation practice: “You’ve got to put your mask on” (P13)

This theme draws on the intersections of race and class and focuses on showing how the institution of rehabilitation as a practice is invested in whiteness by displaying participants' race-conscious awareness in how they navigated rehabilitation, how this shaped rehabilitation goals, and what rehabilitation providers did to support navigating whiteness. All participants brought a race-conscious awareness as part of how they resisted and survived systemic anti-Black racism in the practice of rehabilitation as they understood that it is not a colourblind environment. Survivors understood that their Black identity was a barrier to meeting rehabilitation goals. There were captivating intersectional differences according to class which provided different cognitive strategies to resist and survive whiteness and systemic anti-Black racism in rehabilitation. Participants who were economically disadvantaged had repeated experiences where being Black impacted their care, leading to unfavourable outcomes such as being denied rehabilitation and receiving care of lesser quality. These survivors expressed that they just accepted that their Blackness will always be a barrier to meeting their rehabilitation goals and that they will need to walk away from discriminatory rehabilitation providers but will continue to navigate the system on their own to find the support they need. For example, one woman survivor took matters into her hands and created the rehabilitation she needed,

*“That's why even when I started attending craft programme, because I know craft with the hand and stuff helped my brain. I think that's when I was…l’ll out go out and volunteer at a…let's say I want to do colouring and I know that helped me. And then also, I need social interaction, I can’t be home all the time. So what I'll do is I'll look online for volunteer opportunity, to help with the senior with colouring or activities. And then I'll go and volunteer for that, bringing what I needed to that programme. So at the same time, it's helping me and it's helping them. And then I also created a social programme where people do naming stuff like, time and place orientation. By doing that with a senior is always, it’s like I'm doing everything to…the rehab that I'm not getting, I'm creating it and then implementing it with a senior and then at the same time, I'm getting what I need.”* (P4, Woman, Survivor)

Others relied on their social support networks, such as their friends and family and, in many cases, their Black rehabilitation provider, who helped them navigate the neglect of white clinicians. On the other hand, survivors who were economically privileged pushed back through self-advocacy by selecting their rehabilitation team, which helped to shield away racist encounters but still worked on rehabilitation goals that approximated whiteness.

*“I made it very clear when I was looking for professionals that I preferred women, and I preferred people of colour, because I just find I get better medical treatment, and empathy, and compassion from women, and I also get a greater level of understanding of people who are black or brown… I found a physiotherapist that’s black, and my psychologist is Indian. So, I was able to find a few people who could understand, and the people who aren’t black, they at least are able to be willing to listen to the lived experience of being black, and they have an understanding of the disparities in medicine that occur because I’m black. So that’s the bare minimum…”* (P2, Woman, Survivor).

An important note is that nearly all survivors self-advocated for themselves while in rehabilitation, although there were differences according to how they did this, how much they were heard and the number of times they felt neglected by their rehabilitation providers. However, the institution of rehabilitation is not race-neutral rather, it is set on white norms and white values, for example, one rehabilitation provider shared,

*“They [white rehabilitation providers] don't want to ask the question or get that information because then their job might be a little bit more complicated. When it really isn't going to be, it's just navigating yourself through that person's life and understanding that you are not them and they are not living your life, but you need to live their life the way they are living. You know, what I'm trying to say is there are a lot of – and I see this a lot where if it's a white therapist, whatever the therapist is, they come with their own worldview. And they can't adapt to other people's worldview because they're white, they don't have to, right?”* (P1, Woman, Rehabilitation Provider)

Survivors were aware of that, as a result, they had to carry themselves in particular ways that approximated whiteness. For instance, one male survivor said the following,

*“…A brain injury is probably the last thing a black man wants to get… Because a black man’s life is already complicated, it’s already complicated. You throw in a brain injury there, you start to add in all the other things […] You’re using your brain when you don’t even know it. So, it’s to have all those things altered and then still have to put on this mask, it’s a lot…Because somewhere along the line, your mask isn’t going to be fitting right, you know what I’m trying to say? It’s not going to be fitting right because you got this going on in your brain and you’ve got a whole bunch of whatever chaos going on…my social worker was helping me a bit with my anger as well after. But I think it really comes down to how much the person wants to be involved and engaged in the rehab too. And I think at that point I wasn’t too focused on my mental. I was like, “Get the hell away from me if you’re trying to get into my brain. I don’t really want you around me” …it’s you’re putting in someone who I cannot relate to at all. So, it’s – and I think it also goes back to what I said – you’ve got to put your mask on. You’ve got to prepare yourself a certain way, in order to rise up within the society that’s created in that certain way…I was so focused on the physical aspect of it. So, I was so focused on just trying to get back to soccer, trying to get back to looking good and not looking like I had a brain injury and this and that, that maybe I or maybe they, gave me a short end of the stick on the mental or the cognitive side of it.”* (P13, Male, Survivor).

The excerpt above illustrates how this participant selected rehabilitation goals that he felt were appropriate by emphasizing that *“… you've got to put your mask on. You've got to prepare yourself a certain way in order to rise up within the society that's created in that certain way….”* The mask symbolized that whiteness holds value in rehabilitation and society, reinforcing that Blackness does not. For Black men who have to carry themselves in the world in ways that approximate whiteness as a survival mechanism, the cognitive and physical limitations accompanying TBI make it difficult to wear that "white mask." He uses the metaphor of a white mask [80] to describe how he attempted to approximate whiteness. This displayed his double consciousness [81] by showing how he concealed his true self which meant that he had one mind that he intended for white people to see and another that represented his true self as a Black man but his ability to do this was complicated by the cognitive sequelae related to the TBI. As part of this encounter, P13 described the difficulty in having to put on this “white mask” as a Black man with a TBI. He shared, *“Because somewhere along the line, your mask isn’t going to be fitting right, you know what I’m trying to say? It’s not going to be fitting right because you got this going on in your brain and you’ve got a whole bunch of whatever chaos going on with your mind or whatever, and then you’ve got to put on [white mask]”* (P13, Male, Survivor). He expressed that he operated in a way that approximated whiteness as that was the only option, he felt like he had to get to a place where he could have the best chance at participating in society. For Black men survivors of TBI, the pressures to approximate whiteness meant that they worked on rehabilitation goals that they believed helped them not "look like" they had a brain injury which meant that they focused on physical functioning and often neglected cognitive and psychosocial elements of their recovery. What is also important to note is how P13 expressed that *"… you're putting in someone who I cannot relate to at all…"* suggesting that the White clinician reinforces whiteness and the need to work on goals that assimilate in close proximity to whiteness. This suggests that perhaps working with a clinician of a similar background would allow for opportunities to connect and work on other goals such as addressing mental health and psychosocial functioning. This point of view was expressed by all the survivors.

Survivors and providers described the whiteness infused in rehabilitation practice and the institutional limits of the support that rehabilitation provided Black people with TBI. Most survivors, caregivers, and rehabilitation providers also expressed the neglect of spirituality in rehabilitation and the importance to the journey of the Black patient and their family. Rehabilitation clinicians expressed their awareness of the importance of Black patients with TBI and their caregivers carrying themselves in a way that resembled white norms in the institutional rehabilitation setting. Rehabilitation therapists shared that other rehabilitation providers assumed that *"Black people know how to navigate…through the system, right? Some of them assume it, some of them don't even think about it to be honest with you. And we normally do not do well navigating the system…"* (P1, Woman, Rehabilitation Provider). They described that they helped their patients strategically navigate by helping them be more aware of how their Blackness was perceived by other healthcare providers and by teaching them how to navigate the rehabilitation system. Many attended appointments with their Black clients, and some discussed how they would model those behaviours to learn how to navigate the rehabilitation in a way that would meet their needs because, as they described, the system was not designed with their best intentions at heart. Similarly, caregivers discussed the challenges of approximating whiteness and what they did to support their loved one. For instance, one caregiver shared the following narrative to describe the cost of approximating to whiteness for her husband with TBI,

*“Your skin colour is a weapon. You’re walking in with this loaded weapon, so you need to deescalate the situation. And sometimes that – like his OT for example…the first month she was coming here, she didn’t realize that he was kind of in crisis, mentally; because, again, it was this pleasantness. This little white lady coming into the house, he’s pleasant, he’s respectful. I had to pull her aside and say, “Listen, here’s what he’s going through. Here are the things that I need you to address.” And that wasn’t her fault. She’s a super sweet lady. But he had that wall up to deescalate the situation, to be non-threatening. Make the white lady feel comfortable in the house. You get what I’m saying?* (P10, Woman, Caregiver).

Rehabilitation therapists reported that the one-size-fits-all approach to rehabilitation lacked consideration of the needs of Black TBI patients, how these needs might be different, and the barriers they experienced in participating in meaningful occupations, including mundane activities like going for a walk. A view that all survivors overwhelmingly reported.

### The institutional construction of deficient Black futures: "You know, this is not a crack, this is a massive, gaping hole… some of them feel that there might not be a need to have that discussion" (P14)

This theme focuses on how rehabilitation providers' actions construct deficient Black futures. This theme was organized into three sub-themes which begin with a) lowered expectations of Black patients with traumatic brain injury and b) lack of investment in the futures of Black patients.

#### Lowered expectations of Black patients with traumatic brain injury

One of this study's most striking and alarming findings is the repeated narrative of neglected Black futures. Rehabilitation providers described that Black TBI patients often got pushed out earlier from in-patient rehabilitation and did not have people following up on them to help them figure out what they needed or if they were even being cared about. The institutional construction of deficient Black futures begins with neglecting to have conversations about participation in meaningful occupations for Black TBI patients. For example, a female provider recalled the following encounter while supporting a young Black male with a TBI who wanted to work towards meaningful occupation,

*"…I wasn't there at the beginning, but you've had a team with you. The whole purpose of the team is to walk you through your rehab… and, you know, if you're entitled to this then at this point in time you should not have to be dealing with this right now. This is what you should have had…So I said well, is one of your goals finishing your [general education degree] GED? Because you're young, you're going to live for a long time, what are your plans? Nobody has really addressed those issues with him. He's got a whole team that just seem to be dealing with the here and now. And I'm going he's young, he's 18. Why are we not talking about things that will give him some kind of life as he gets older? He then tells me he was a drop out, he was a criminal, he was actually on bail, on parole when he had his accident, and I'm going so how come nobody knew this? How come nobody knew this? How come we haven't been addressing his future? Like it didn't matter. And I've had people who've had less of a future than him and everybody's gung-ho trying to them this and get them that and, you know. And I'm going this is ridiculous, right? Why does it take a physio – this is not even my job and he's had an OT with him for the two years that I didn't see him..."* (P1, Woman, Rehabilitation Provider).

This above excerpt not only suggests that the occupational therapist working with this young Black male likely did not complete an occupational profile asking important questions about his interests, roles, how he likes to spend his time, what the barriers are to doing those meaningful activities of daily living, and what his priorities are, but also that the rehabilitation team did not take the time to get to know him as a human with a life worth living. The fact that nobody knew about how he was someone who was interacting with the criminal justice system and likely still is despite working with a rehabilitation team for two years is disheartening and concerning. Given that the goal of rehabilitation is to maximize functioning to support participation in activities of daily living, it is questionable what the rehabilitation team of therapists was doing with the clinical hours they spent with him. The excerpt above illustrates the rehabilitation teams’ lowered expectations for this young Black male and why Black rehabilitation providers frequently felt they were responsible for ensuring they look out for Black TBI patients.

#### Lack of investment in the futures of Black patients

Rehabilitation providers expressed that while the health care system relied on best practice guidelines that did not consider the needs of the populations they served, this issue was much deeper. The narratives from clinicians revealed that Black TBI patients would not receive the same standardized assessment in the same manner as White TBI patients. They described that White rehabilitation providers held lowered expectations for Black TBI patients. Their experiences of working with other clients prompted them to notice that Black TBI patients often did not receive any vocational assessments, but this was commonly present in the treatment plans of White patients. For example, P14 shared,

*“… I don’t recall any of my black clients actually having the vocational rehab assessment. It was more, that service was more given to White clients… So there as a sense of looking toward gainful work, potentially paid work and reintegrating into a working environment at some future point, that it was considered something that was going to be part of their goals moving forward. And for those particular clients it really wasn’t, it wasn’t questioned really. It was like this, this was built into their rehab plan…”* (P14, Woman, Rehabilitation Provider).

The example above suggests that Black TBI patients are not seen as people who are worthy of participating in productive activities or have anything to offer in contributing to society. While many believed that having access to funding would provide the opportunity to have resources to think about the rest of one’s life, our analysis showed otherwise. Funding and access to a rehabilitation team do not always mean equal opportunities to engage in meaningful opportunities and think about the rest of one's life. The interviews of other rehabilitation providers revealed that economic privilege did not always equate to equal treatment. Many Black people were robbed of services that they should have received in earlier phases of their rehabilitation journey.

The lowered expectations of and lack of investments in Black TBI patients in the context of the rehabilitation setting continues when they transition out of rehabilitation and into the community. The interviews of participants revealed that community integration continues to be a significant challenge for Black TBI survivors. Black male survivors of TBI expressed that rehabilitation was only able to do so much to support the needs of a Black man with a TBI and the challenges of integrating into society,

*“… there were other areas in which I did not receive the help in which I needed, or, you know, rehab wasn’t really ... Yeah, they weren’t able to provide that sort of help ultimately because I was a Black individual. And I face various obstacles and just in society, you know, and throughout life…because there are certain things that rehab –like they could help you with certain things, but day-to-day experience, you know, everybody has to go through their own journey pretty much, right?”* (P15, Man, Survivor).

Additionally, both Black men and women survivors expressed that rehabilitation did not give them the tools to navigate social interactions in different environments in the workplace. Their narratives revealed that they were not always encouraged to engage in social communication with their peers or how to navigate communication barriers during rehabilitation resulting in lost friendships, family members, and opportunities to participate in productive employment. Together, this displays the institutional construction of deficient Black futures.

## Discussion

This critical narrative inquiry is the first study to consider the Black experience of TBI in rehabilitation across the care continuum in Canada and includes the perspectives of Black survivors, rehabilitation providers, and family caregivers. The findings from this study illustrate how the actions and decisions of rehabilitation professionals determined care outcomes and life experiences, holding material consequences for Black people with TBI and filling several gaps. These gaps include documenting the experiences of Black people with TBI and an analysis that identifies how racism becomes institutionalized in rehabilitation practice. Four themes were conceptualized, which identified that racism manifests in rehabilitation institutions through (a) the institutional construction of deficient Black bodies, (b) the institutional construction of rehabilitation access, (c) the institutional investments in resisting and approximating whiteness in rehabilitation practice, and (d) the institutional construction of deficient Black futures. Four tenets of CRT were particularly pertinent to this study in identifying how racism becomes institutionalized in TBI rehabilitation practices, policies, and procedures. In this section, we intend to integrate these tenets into the themes conceptualized from the analysis and discuss implications for practice, research, and education. A brief discussion of the strength and limitations and the broader implications of this inquiry will follow.

### Racism is an ordinary experience in traumatic brain injury rehabilitation

CRT centers on the premise that racism is pervasive, an integral part of society, and the ordinary way institutions operate [48, 49] such as TBI rehabilitation. One mechanism of the ordinariness of racism is through practices and procedures that promote a false illusion of fair and equal treatment that instead function to perpetuate whiteness and maintain White supremacy in rehabilitation using ideologies such as colourblindness (e.g., neutrality) and meritocracy (e.g., hard work and merit) which minimize the significance of race. This is seen in participant narratives where they bring a race-conscious awareness to rehabilitation by using different strategies afforded by class privilege to resist whiteness and anti-Black racism and, in other instances, approximate whiteness to benefit from rehabilitation. Other literature has shown that racism and whiteness operate to provide White patients with more appropriate health care and that providers in medicine, nursing, and dentistry provide care informed by colourblind ideologues [82–84]. The findings from this study support this premise and provide important nuances relevant to a rehabilitation context.

An important finding is the use of the metaphor, "white mask," which was coined by Franz Fanon [80]. This metaphor referred to the kind of code-switching that happened for Black men with TBI where the white mask represented the need to assimilate to the norms and values of whiteness while in a rehabilitation context. In *The Souls of Black Folk,* CRT scholar Du Bois [81] used the term double consciousness to articulate "this sense of always looking at one's self through the eyes of others" (p.10-11). For instance, double consciousness was prevalent in survivors' narratives, demonstrating that they came into TBI rehabilitation and realized that to benefit from services offered and receive optimal care, they needed to operate in a way that approximated whiteness which was not only difficult but also had its consequences. This meant that Black men worked on rehabilitation goals that a White clinician could understand, like addressing physical functioning and neglecting cognitive and emotional well-being (e.g., mental health), which raised further challenges on their road to recovery. This may result in what is referred to as the 'burden of acting white hypothesis' seen in Black students across different academic settings leading to underachievement [85–88] raising critical questions about the types of goals rehabilitation providers recommend, encourage, and support. TBI rehabilitation must understand the costs of double consciousness and address efforts towards eliminating the need for Black patients to feel that they need to conform to a particular set of standards defined by race if the goal of rehabilitation is to maximize functioning and participation in daily activities for all people.

Black TBI patients may benefit from particular rehabilitation programs and services that are intentionally designed to give them the tools they need to counter the kinds of realities faced by Black people when participating in society (e.g., categories of skills to work on, types of meaningful occupations to engage, how to meet participation goals). This does not take away from the need for rehabilitation professionals who can appreciate and understand the complexities and nuances of the lived experiences of Black people and how that may be used in a rehabilitation context to provide the space to work on holistic goals such as those related to cognition and mental health. This may also call for interdisciplinary collaborative healthcare to support psychological goals such as identity reconstruction and affirmation post TBI. These findings have important implications for anti-racist rehabilitation (e.g., rehabilitation that challenges whiteness and White norms), which is needed if rehabilitation is meant to support all persons with TBI. Perhaps professional rehabilitation education can focus on creating the next generation of anti-racist rehabilitation professionals to help improve quality of life.

### Social construction of deficient Black bodies with traumatic brain injury

One of the hallmarks of CRT is the fact that race is a social construct with no biological basis and was created to legitimize the subordination of Black people and other racialized persons [89, 90]. For instance, the dominant narrative in research about race and TBI rehabilitation often portrays a deficit perspective about Black people, continuously describing them as experiencing worse outcomes across various measures compared to their White counterparts. This is seen in the institutional construction of deficient Black bodies. Our findings provide deeper insight into how deficient views operate in a rehabilitation context spotlighting essential nuances related to intersections of race and gender and the consequences. When Black men with TBI enter rehabilitation settings, rehabilitation professionals and other care providers treat their Blackness as the presenting problem rather than addressing the clinical manifestations of the injury. This is seen where Black men with TBI are labelled as aggressive, violent, and dangerous, unlike their White counterparts, which is similar to the kinds of dominant narratives perpetuated about Black men that have been well documented [91–94].

Similarly, Black women caregivers were labelled as being complicated and problematic when advocating and supporting their loved ones compared to White caregivers. This can be recognized as the trope of the angry Black woman stereotype which casts Black women as aggressive, ill-tempered, illogical, overbearing, hostile, and attitudinal [95, 96]. This reinforces the importance of considering race and gender in addressing these racist outcomes in rehabilitation practice. Addressing institutional racism should involve monitoring what kinds of narratives get documented about Black people with TBI and how this shapes referrals to rehabilitation services and programs.

Applying CRT brings our attention to the norms of whiteness and how it sets the standards [49], in this case, the ideal White patient and the problematic Black patient. Frankenberg [97] describes that the white racial frame makes whiteness normative and the unspoken standard by which all other people are judged and viewed. This white racial frame may account for why White TBI patients were tolerated and treated with dignity and Black TBI patients were treated as problematic unrelated to their injuries. These findings are significant as they suggest that rehabilitation can be a dangerous place for Black people with TBI, with gendered consequences as the white racial frame constructs Blackness as synonymous with criminality (e.g., narratives of violence, aggression, drug users, drug dealers) which reinforces racial profiling and becomes more dangerous the more significant the severity of the injury is and if the injury is sustained through mechanisms of violence. This can impact how Black TBI patients are orally presented during clinical meetings, the kinds of information shared about them, and the stories shared between service providers. In this way, other service providers may refuse to work with them and see them as problematic, leading to a domino effect which can result in discontinued rehabilitation, reduced hours in rehabilitation, lowered quality of care, unsuitable treatment options, and lack of referrals and recommendations for continued care. If those narratives get written into their medical records, then that becomes the story people tell about them, which means that opportunities for recovery and rehabilitation become limited.

These findings are significant as these written stories can reinforce conscious and unconscious beliefs about Black TBI patients and who deserves to benefit from rehabilitation. This could contribute to different outcomes based on race, such as poor functioning, participation, integration, and long-term outcomes which are then reproduced in research. Addressing conscious and unconscious bias is only one part of the more significant issue of institutionalized racism in TBI rehabilitation. Our findings reinforce the fact that racism operates on multiple levels which need to be addressed, including interpersonal (e.g., conscious and unconscious bias, clinician-patient) and institutional (e.g., unfair policies, practices, discriminatory treatment, inequitable opportunities). There is a serious need to identify how rehabilitation institutions can hold clinicians responsible for the clinical decisions made and their consequences. Future research is needed to identify ways to implement accountability measures in practice.

### Traumatic brain injury rehabilitation is white property

One of the major tenets of CRT, which was crucial in understanding the cumulative impacts of institutional racism in TBI rehabilitation, is the tenet of whiteness as property which refers to rights based on whiteness [51] as illustrated in Fig. 1. One of these rights is the right to exclude [51], and meritocracy is one mechanism used to exclude Black people from TBI rehabilitation. In CRT, meritocracy, which allocates resources and opportunities, is inherently inequitable as it fails to account for the systems and institutions that exclude racialized people [49], and in this case, Black people with TBI. Meritocracy enables more White people to gain access to rehabilitation despite the illusion that access depends on funding, injury severity, and other objective criteria. However, meeting these merit-based requirements is increasingly difficult for Black people with TBI. It is important that rehabilitation institutions keep track of whether the number of Black people entering into rehabilitation programs and services is increasing or decreasing.

This study draws attention to how rehabilitation therapists gatekeep [98] services, resources, and knowledge at the level of both publicly funded services and through insurance companies in the province of Ontario. As similarly reported in previous studies [8, 9, 99], Black survivors in this sample overwhelmingly experienced challenges getting into rehabilitation where some have expressed waiting for many years, including up to a decade. These decisions are made at the professional discretion of the rehabilitation professional and their clinical reasoning. What is quite interesting is at the level of insurance companies where participants revealed how rehabilitation professionals used race as part of their assessments to determine whether or not Black TBI patients' injuries were worthy of rehabilitation which may also explain why Black patients with TBI have difficulty accessing rehabilitation even when covered by insurance [7, 11]. The literature has shown that Black people with TBI are less likely to get into rehabilitation even after controlling for injury severity and insurance status [99] and are more likely to get discharged home [9]. Although it is beyond the scope of this study to go into a lengthy discussion about ethical and clinical reasoning, it is important to mention that perhaps what underpins profession-specific rehabilitation models and theories may contribute to and reinforce the poor outcomes that disproportionately impact Black TBI patients in rehabilitation that warrant further investigation. Future researchers may also wish to consider examining the process of rehabilitation admittance through public and private services and identify the interplay between racially biased standardized questions and subjective questions spontaneously created by the assessor. Equally important is examining the intake process, clinical decision making, and what criteria rehabilitation professionals use to determine who gets into rehabilitation and who does not. Over time, rehabilitation institutions must monitor how the number of Black TBI patients in rehabilitation compare to the number of White patients.

One of the most demoralizing findings from this study is the construction of deficient Black futures, which illustrates a deeply rooted issue in society that impacts all Black people generally, specifically those with TBI which is seen even in the education system for Black people with TBI [100] and other Black students who are denied opportunities to learn and participate in academic life [101–103]. When rehabilitation providers neglect to ask questions about participation and community integration, disregard the need to provide necessary treatment, and differentially incorporate pertinent assessments, it reinforces rehabilitation as a property owned by and for the use of White patients and other White people. In this context of rehabilitation practice, whiteness as property refers to the fact that whiteness as an identity is the acceptable standard and norm that is depicted to be the beneficiary of rehabilitation support. This means that Black patients are not afforded the same rights in being supported in occupations, having opportunities to participate in community life and the rights to benefit from rehabilitation as their White counterparts. Rehabilitation institutions must audit the treatment plans of Black TBI patients in rehabilitation to monitor what kinds of recommendations, referrals, assessments, and goals Black patients are supported with which can also function as an accountability measure for how conscious and unconscious racialized beliefs about Black people’s worth and value in society contribute to lower expectations and the lack of investments in the futures of Black people with TBI.

What if rehabilitation practice made a conscious effort to invest in Black patients' futures? Future research may wish to examine what this could look like given the findings of this study. It is important to note that this does not mean that Black people with TBI do not benefit from rehabilitation, but that practices, procedures, and policies were not designed for their benefit. Rehabilitation professionals put less effort into thinking about the futures of their Black TBI patients by not engaging in discussions about how to support participation in vocation and other meaningful occupations, which appeared to be the standard treatment for all White patients. Further, economic privilege does not insulate Black survivors from the realities of not being seen as worthy enough to be considered for opportunities to engage in meaningful employment and other productive activities, including going to school and participating in community life. Similar to the participant narratives, other studies using different methodologies and based in the U.S. have shown that Black persons with TBI receive less intense treatment [10] and experience poor community integration and participation in productive activities [13–16, 104, 105]. Perhaps this is why Black people with TBI spend more time unemployed. Future research should consider important components needed in rehabilitation programs and services to dismantle whiteness (e.g., supporting alternative ways knowing, doing, and being) and support participation in daily life for Black survivors of TBI. This should also involve examining what discharge planning looks like for Black people and ensuring that they have connected supports when transitioning into the community.

## Strengths and limitations

One of the strengths of this inquiry is the triangulation of three perspectives in understanding how racism operates in TBI rehabilitation practice. The sample of participants reflected a diverse range of perspectives from the spectrum of TBI severity, lived experiences, mechanisms of injury, class, and gender which provided a range of knowledge from where investments in Black futures need to be made. The breadth of questions in this study and the data collection methods resulted in a wealth of knowledge about the Black experience of TBI in rehabilitation and across the life journey. This study demonstrates how CRT can help understand Black experiences in TBI rehabilitation. Despite efforts to maximize variation, participants were a specific group of people who were capable of participating and interested in Blackness as part of their rehabilitation experiences, which means that the findings must be viewed in that perspective. Since all participants from this study were residents of the province of Ontario, replicating these findings across Canada can help better understand Black people's experiences with TBI across different jurisdictions and provinces, as some jurisdictions do not have fault claims for motor vehicle collisions. Importantly, including a gender-diverse sample is also important in capturing nuances in perspectives. Future studies should also consider perspectives from other rehabilitation professions, such as physiatry and speech-language pathology. Future studies may also benefit from using other methods of inquiry such as mixed methods to gather information about other dimensions of this population's needs. More research is needed to improve outcomes and quality of life, such as improving rehabilitation services and developing tailored interventions and programs for this population to support living a meaningful and fulfilling life. Although the sample size was small, the findings are rich and nuanced and reveal important information about the nature of institutional racism in TBI rehabilitation practice, which require immediate action across the Canadian healthcare system and beyond.

## Conclusion

The findings from this study identified four themes capturing how institutional racism manifests in rehabilitation practice for Black people with TBI. These themes addressed the institutional construction of deficient Black bodies, institutional gatekeeping of rehabilitation services, institutional investments in promoting whiteness, and institutional construction of deficient Black futures that carries over into poor transitions into the community. The power of institutional racism is that a single person does not have to carry racist views or intend to provide racially inequitable care. Suppose rehabilitation as an institution was not designed with Black bodies in mind, and the research informing the practice is inherently biased toward producing deficient narratives, narratives about racial disparities, and rehabilitation therapists shape deficient futures. What does that say about the institutional space of rehabilitation? By conducting this study, we invite other researchers to consider how institutional racism shapes rehabilitation goals, trajectories, outcomes, and opportunities to participate in meaningful occupation. We hope that future research considers what it takes for Black patients with TBI to have a life worth living under these conditions while in a rehabilitation institution supposedly designed to help the patient heal and maximize functioning to participate in community life.

### Supplementary Information

**Supplementary Material 1.** 

## Data Availability

Due to ethical restrictions, data are available from the corresponding author on reasonable request.

## Abbreviations

CRT: Critical race theory
CDC: Centers for Disease Control and Prevention
OSU TBI-ID: OHIO State University Traumatic Brain Injury Identification Method
PHAC: Public Health Agency of Canada
TBI: Traumatic Brain Injury
U.S.: United States
