# Supplementary material for: “Somewhere along the line, your mask isn’t going to be fitting right”: institutional racism in Black narratives of traumatic brain injury rehabilitation across the practice continuum
Source: BMC Health Serv Res. 2024 Jul 24;24:834. doi: 10.1186/s12913-024-10986-1 (PMC11270842; doi:10.1186/s12913-024-10986-1)
Supplement: Supplementary file 1 — Supplementary Material 1. [file 12913_2024_10986_MOESM1_ESM.pdf]

Additional File 1 – Interview Guide 1 for Black Survivors of Traumatic Brain Injury

Study title: Amplifying Black Voices in Traumatic Brain Injury Rehabilitation: A Critical Examination of the Narratives of Black Survivors and their Caregivers

Welcome, and thank you so much for participating in this research study about Black peoples' experiences in traumatic brain injury rehabilitation.

As you know, the purpose of this research study is to understand the voices and perspectives of Black people who live with or care for someone with a traumatic brain injury and how they narrate their experiences. To do this, I will first begin to ask you some demographic questions which will then be followed by some open-ended questions down below. You are welcome to answer any way that you like and you may choose to not answer a question if you are uncomfortable. Your participation in the study is voluntary and you can withdraw from the study at any time without any consequences to your care, services, and/or employment.

I want to acknowledge that I am aware that some people who have experienced a traumatic brain injury may also have difficulty with cognitive tasks like memory, planning daily activities, multi-tasking, concentration, and physical challenges such as headaches, dizziness, eyesight, and ringing in their ears. They may also feel they have trouble managing their emotions or feel depressed or anxious a lot of the time. Take your time. There is absolutely no rush.

In order to ensure accuracy for the data analysis, I will be audio-recording this interview. I will let you know when we start recording. There are no right or wrong answers and you are free to refuse to answer any questions that you don't feel comfortable with. You decide how much you want to share in this space. Please also know that you can take a break or stop the interview at any time. All you have to do is just let me know. Do you understand your participation in this study? Do you have any questions before we begin?

[I will now begin recording]

1. Can you please tell me in as much detail as you can your story of what it is like to be Black (man or woman) who is living with a traumatic brain injury and your experiences with rehabilitation? Please feel free to begin wherever you like. If it is helpful to you, you can refer to memories before your traumatic brain injury, you can also share how you experienced your traumatic brain injury to help share your experience, how the traumatic brain injury may have changed how you participate in the activities that you need, want, and love to do in your life. There are no right or wrong answers, this is your unique story.

Narrative Prompts Plus

2. Did you and/or do you currently notice any ongoing challenges with any of the following after your brain injury?
   Please circle:

Headaches	**Y**	**N**	Trouble concentrating	**Y**	**N**
Memory problems	**Y**	**N**	Depression or Anxiety	**Y**	**N**
Dizziness	**Y**	**N**	Difficulty multi-tasking	**Y**	**N**
Ringing in your ears	**Y**	**N**	Problems organizing tasks	**Y**	**N**
Fatigue or tiredness	**Y**	**N**	Mood swings or anger	**Y**	**N**
Sleep problems	**Y**	**N**	Light or sound sensitivity	**Y**	**N**
Loss of taste or smell	**Y**	**N**	Hard to follow conversations	**Y**	**N**
Flashbacks	**Y**	**N**	Nightmares	**Y**	**N**

   Other:____

3. Can you tell me about what activities you love, need, and want to do in your life?
   a. What does it look like to have a social support network?

4. As a Black person with a traumatic brain injury, how do you feel rehabilitation supported your needs to participate in everyday living inside and outside rehabilitation?
   a. Who supports you with assistant in your everyday activities if you need it?
   b. Can you tell me about a time where you felt like you were supported in participating in activities that are important you?

5. How does your identity as someone who is Black impact your ability to participate in your day to day activities inside and outside rehabilitation?
   a. As a Black [insert gender] experiencing a traumatic brain injury, what challenges have you experienced inside and outside of rehabilitation?
   b. How do you cope with the challenges that you may experience as a result of your traumatic brain injury?
6. Can you tell me about a time where you experienced racial microaggressions as a caregiver for someone who is experiencing a TBI? (racial microaggressions are the types of questions or comments that are insulting and painful because they have do with our race or ethnicity)
   a. A time where you felt minimized because of your identity as someone who is Black?
   b. How did you make sense of why you were being treated differently?
   c. How did you deal with this?

7. Can you tell me about a time where you felt discriminated against or treated differently in rehabilitation because of your race, ethnicity, faith, gender, sexuality, and or disability?
    a. A time where you were made invisible in rehabilitation and outside rehabilitation?
    b. How did you make sense of why you were being treated differently?
    c. How did you deal with this?
    d. Are there other aspects of your identity that have shaped and continue to shape your experiences?

8. How has your socioeconomic status (income, education, and occupation) impacted/shaped your rehabilitation experience as a survivor of traumatic brain injury?
    a. If you received rehabilitation services outside of a hospital, how do you cover the costs of the rehabilitation services you received?

9. How have things changed for you during the COVID-19 pandemic?
    a. How has the pandemic changed the way that you participate in everyday activities for the better?
    b. How has the pandemic changed the way that you participate in everyday activities for worse?

10. If rehabilitation could meet your unique needs as someone who is Black and experiencing a traumatic brain injury, what would it look like?
    a. What would it consist of?
    b. What does it mean to be living a good life?

<u>Non-Directional Probes</u>

That's interesting. Can you tell me more about that? What do you mean when you said that? What happened when you said? What was the experience like for you?

Thank you so much for taking the time to participate in this interview. I am now going to turn off the recorder.
